# Supplementary material for: Influence of Heparan Sulfate Proteoglycans and Factor X on species D Human Adenovirus Uptake and Transduction
Source: Viruses. 2022 Dec 24;15(1):55. doi: 10.3390/v15010055 (PMC9866072; doi:10.3390/v15010055)
Supplement: Supplementary file 1 [file viruses-15-00055-s001.zip › viruses-2100394-supplementary.pdf]

Supplementary Material

Method M1: Details on the determination of the HSPG-FACS results

For analysis of the HSPG-positive cells, an isotype control (Iso, only the secondary antibody was used) and cells incubated with anti-HSPG antibody were used. The used first antibody was a mouse anti-human heparan sulfate IgM (10E4 epitope, clone 8.S.087) which detects only sulfated forms of heparan chains. Therefore, the CHO-K1 cells showed 99% positive cells and the CHO-606 and -745 only 12%. As a secondary antibody, goat anti-IgM to mouse with AlexaFluor® 488 was used. The data was analyzed by first using sideward and forward scatter gating from the Isotype control to exclude cell debris. Stained cells were then gated in a histogram with FITC channel (on x-axis in log displayed, for AlexaFluor® 488 detection) against the cell number (y-axis). In the analysis tool of the software, an overlay of the isotype and the anti-HSPG diagram was generated which is shown in Figure 2B in the main manuscript.

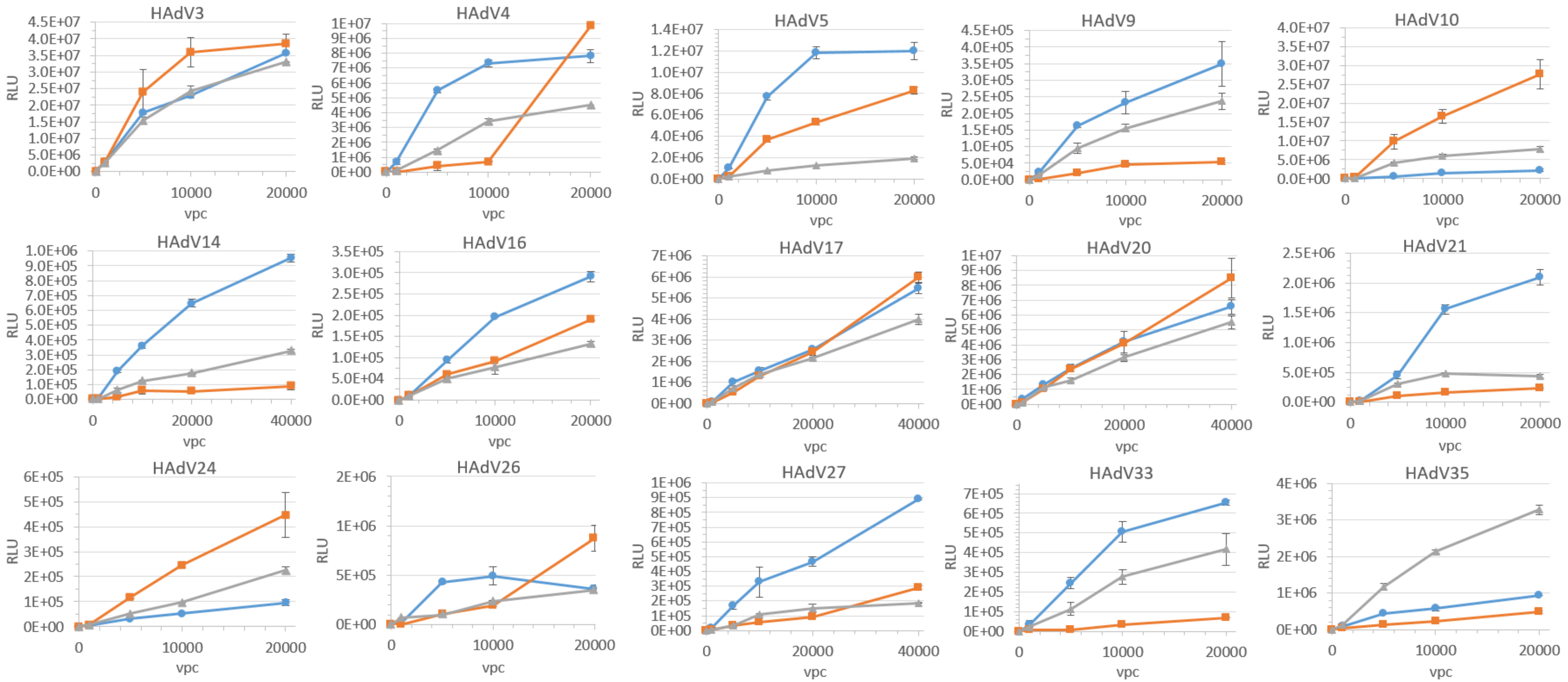

Figure S1. GLN-tagged adenovirus library screening in HSPG-depleted CHO cell lines. 24 h post-infection, with 1,000 to 10,000 vpc (viral particles per cell) luminescence was measured. The results are displayed as RLU (relative light units). Normal standard deviation (SD) was used. In blue (circles) are the results from CHO-K1, in gray (triangles) from CHO-606 and in orange (squares) from CHO-745 shown. Displayed is one out of three independent experiments for each virus used.

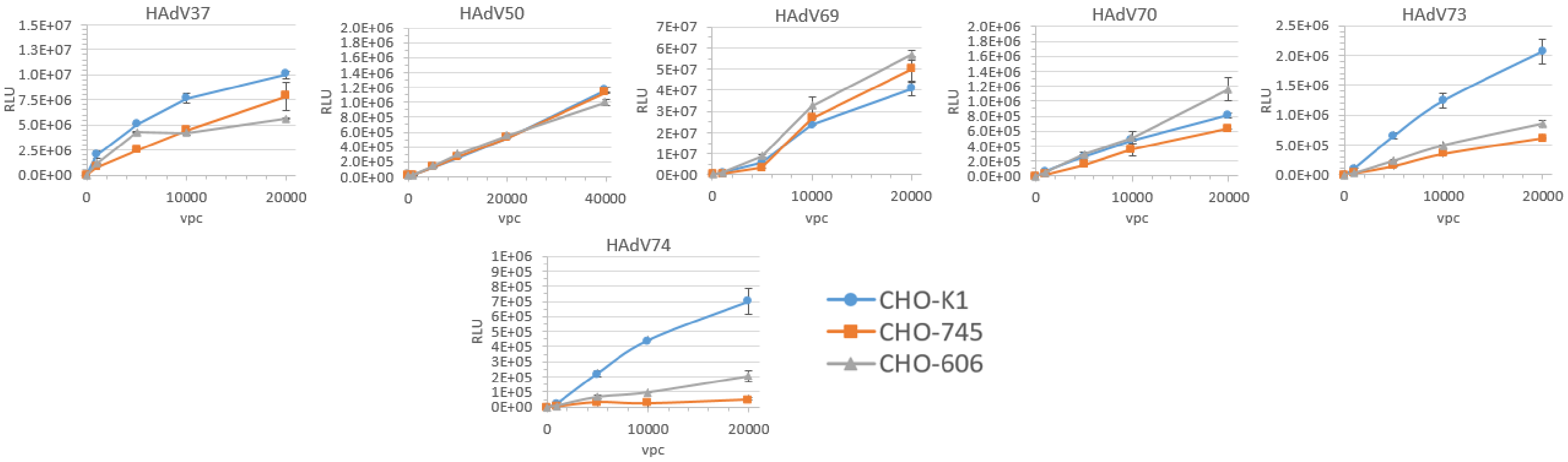

Figure S1. Continued.
